# Increased p66Shc in the Inner Ear of D-Galactose-Induced Aging Mice with Accumulation of Mitochondrial DNA 3873-bp Deletion: p66Shc and mtDNA Damage in the Inner Ear during Aging

**DOI:** 10.1371/journal.pone.0050483

**Published:** 2012-11-27

**Authors:** Lisa Wu, Yu Sun, Yu-Juan Hu, Yang Yang, Ling-Li Yao, Xing-Xing Zhou, Hao Wang, Rui Zhang, Xiang Huang, Wei-Jia Kong

**Affiliations:** 1 Department of Otorhinolaryngology, Union Hospital, Tongji Medical College, Huazhong University of Science and Technology, Wuhan, People’s Republic of China; 2 Department of Otolaryngology-Head and Neck Surgery, Xiangya Hospital, Central South University, Changsha, People’s Republic of China; 3 Institute of Otorhinolaryngology, Union Hospital, Tongji Medical College, Huazhong University of Science and Technology, Wuhan, People’s Republic of China; 4 Key laboratory of Neurological Disease, Ministry of Education, Wuhan, People’s Republic of China; University of Texas Health Science Center at San Antonio, United States of America

## Abstract

Aging has been associated with mitochondrial DNA damage. P66Shc is an age-related adaptor protein that has a substantial impact on mitochondrial metabolism through regulation of the cellular response to oxidative stress. Our study aimed to establish a D-galactose (D-gal)-induced inner ear aging mouse model and to investigate the potential role of p66Shc and its serine 36-phosphorylated form in the inner ear during aging by using this model. Real-time PCR was performed to detect the mtDNA 3873-bp deletion and the level of p66Shc mRNA in the cochlear lateral wall. Western blot analysis was performed to analyze the total and mitochondrial protein levels of p66Shc and the level of Ser36-P-p66Shc in the cochlear lateral wall. Immunofluoresence was performed to detect the location of the Ser36-P-p66Shc expression in the cochlear lateral wall. The results showed that the accumulation of the mtDNA 3873-bp deletion, total and mitochondrial protein levels of p66Shc and level of Ser36-P-p66Shc were significantly increased in the cochlear lateral wall of the D-gal-treated group when compared to the control group and that Ser36-P-p66Shc was mainly localized in the cytoplasm of the cells in the stria vascularis. During aging, the oxidative stress-related increase of p66Shc and Ser36-P-p66Shc might be associated with the accumulation of the mtDNA 3873-bp deletion in the inner ear.

## Introduction

Age-related hearing loss (ARHL) or presbycusis is the most common sensory deficit in the elderly, and it has become a severe social and health problem. Although many theories underlying presbycusis have been proposed, the overall mechanisms remain speculative. Among these theories, many studies support the idea that a mitochondrial malfunction plays an important role in presbycusis [1]–[3]. The mitochondrion is the organelle that generates the high energy intermediate ATP and is also a major source and major target of reactive oxygen species (ROS) [4], [5]. ROS that originates from the mitochondrial respiratory chain can damage macromolecules, especially mitochondrial DNA (mtDNA) [6]. Mammalian mtDNA is a closed circular molecule of approximately 16 kb and encodes many critical components of the oxidative phosphorylation pathway, which is responsible for generating ATP for the cell. Thus, a deletion in the mtDNA would lead to a mitochondrial malfunction. The 3867-bp deletion in mice that corresponds to the mtDNA 4977-bp deletion in humans and the mtDNA 4834-bp deletion in rats, which are also called common deletions (CD), is the most common mtDNA damage associated with aging [7]–[9]. According to the NCBI reference sequence for mtDNA (NC_005089.1), there are 6 bp added to the mtDNA 3867-bp deletion. Thus, it is more accurately a mtDNA 3873-bp deletion. The mtDNA 3873-bp deletion occurs within nt9089– nt12961 or nt9104– nt12976, which are flanked by a 15-bp direct-repeat sequence (AGCCCTACTAATTAC), and results in a partial to complete loss of the following mtDNA genes coding for components of oxidative phosphorylation (OXPHOS): COX3, ND3, ND4L, ND4, ND5 and 5 tRNAs (tRNA-Gly, tRNA-Arg, tRNA-His, tRNA-Ser and tRNA-Leu). Previous studies have suggested that the mtDNA deletion is associated with pathological disorders of presbycusis in human beings, rats and mice [10]–[13]. We have previously utilized overdoses of D-galactose (D-gal) to induce an aging model of rats, which harbor increased amounts of the mtDNA 4834-bp deletion in the central and peripheral auditory system [14]–[19]. Moreover, we found that below a certain level, the mtDNA 4834-bp deletion might not directly lead to a hearing impairment but rather act as a predisposing factor that can greatly enhance the sensitivity of the inner ear to aminoglycoside antibiotics [17]. These findings suggest that the mtDNA deletion plays an important role in presbycusis. As the inner ear tissue of live humans is inaccessible and the genetic and environmental background of individuals with hearing loss is nonhomogeneous, studies on presbycusis are limited. The mouse is one of the mammals, after humans, whose genome has been sequenced. Additionally, approximately 99% of human genes have homologs in the mouse [20]. Thus, compared to the D-gal-induced aging rat, the D-gal-induced aging mouse would provide a more ideal model to explore the potential mechanisms involved in inner ear aging.

**Table 1:** The ABR threshold (dB SPL).

Frequency	NS group (n = 10)	LD group (n = 10)	HD group (n = 10)
4 kHz	39.0±3.94	38.5±5.80	38.0±6.75
8 kHz	30.0±4.83	30.5±5.50	27.5±4.86
16 kHz	23.0±4.22	23.0±4.83	22.5±4.25
32 kHz	34.5±4.38	37.0±4.25	36.0±3.16

The data are expressed as mean±standard deviation. (NS, the group treated with normal saline; LD, the group treated with 800 mg/kg D-gal; HD, the group treated with 1000 mg/kg D-gal. p>0.05 when compared to the NS group.).

The p66Shc protein is a Src homologue and collagen homologue (Shc) adaptor protein, which has the ability to interact with other proteins that mediate cell signaling [21]. It is encoded by the mammalian shcA gene, which also encodes the two other ShcA adaptor proteins p46Shc and p52Shc that are named for their molecular masses [22]. However, only the p66Shc isoform participates in mitochondrial ROS generation and acts as an oxidative signal regulator, which translates oxidative signals to the mitochondria [23], [24]. P66shc-null mice display an increased lifespan and enhanced resistance to oxidative stress [25]. Upon oxidative stress, p66Shc is phosphorylated at a serine residue (Ser36), and the phosphorylation of Ser36 is necessary for the cellular response to oxidative stress [25]. A portion of p66Shc partially localizes within the membrane space of the mitochondria where it oxidizes cytochrome c (Cytc), thus producing ROS [23]. Since the publication by Migliaccio, the p66Shc protein has been recognized as an important element of the free radical theory of aging [24], and many age-related diseases were suggested to associate with p66Shc [26]–[29]. However, no study of p66Shc in presbycusis has previously been reported.

As the stria vascularis is an essential structure of the inner ear that contains abundant mitochondria and has the highest rate of aerobic oxidation in the inner ear [30], [31], we focused on the cochlear lateral wall as the major subject of our study. We established a D-gal-induced aging mouse model that harbored increased levels of the mtDNA 3873-bp deletion in the cochlear lateral walls and investigated the total and mitochondrial levels of p66Shc and the Ser36-phosphorylated form of p66Shc (Ser36-P-p66Shc) in this aging model. Our results suggested that p66Shc and Ser36-P-p66Shc might play a critical role in inner ear aging and the formation and accumulation of mtDNA damage in the inner ear.

**Table 2:** The plasma SOD activity and MDA levels.

	NS group (n = 6)	LD group (n = 6)	HD group (n = 6)
T-SOD (U/ml)	198.62±10.35	170.67±19.11**	146.52±13.42**^#^
MDA (µmol/ml)	1.22±0.20	2.52±0.49**	3.25±0.47**^∧^

The data are expressed as the mean±standard deviation. (NS, the group treated with normal saline; LD, the group treated with 800 mg/kg D-gal; HD, the group treated with 1000 mg/kg D-gal. **, p<0.01 when compared to the NS group; ^#^, p<0.05 when compared to the LD group; ∧, p<0.01 when compared to the LD group.).

## Materials and Methods

### Animals and Treatment

One hundred and two male Kunming mice (median body weight of 22 g; 7–8 weeks old) with normal hearing were purchased from the Laboratory Animal Center of Tongji Medical College of Huazhong University of Science and Technology. The Kunming mouse strain is an outbred strain of the Swiss mouse strain, which came to Kunming, China in 1944 from the Indian Haffkine Institute, and was widely used for the D-galactose-induced aging mice [32]–[36]. The mice were acclimated to the new environment (23°C; 12-h light/dark cycle) for at least one week before the start of the experiments. All the animals had free access to water and food. The animals were randomly assigned to three groups (n = 34 for each group) depending on the dose of D-gal (Sigma Chemical, St. Louis, MO), as follows: a low dose (LD) group, high dose (HD) group and control group. Mice were treated with a daily subcutaneous injection of the D-gal solutions (LD group, 800 mg/kg of D-gal; HD group, 1000 mg/kg of D-gal) or an equal volume of normal saline (NS) (for control group) in the neck for 10 weeks. The animal studies were performed in accordance with the guidelines for the care and use of laboratory animals that were prepared by the Institution of Laboratory Animals of Huazhong University of Science and Technology. The protocol was approved by the Committee on the Ethics of Animal Experiments of Huazhong University of Science and Technology (Permit Number: S244). All surgeries were performed under a mixture of ketamine and chlorpromazine anesthesia, and all efforts were made to minimize suffering.

### Auditory Brainstem Response

At the beginning and at the end of the treatment, the hearing functions of the mice were tested by auditory brainstem responses (ABR). Mice (n = 10 for each group) were anaesthetized with a mixture of ketamine (120 mg/kg) and chlorpromazine (20 mg/kg) and placed on a heated mat in an audiometric chamber. The sound delivery tube of the insert earphone was tightly fitted into the external auditory canal. Electrodes were placed subdermally over the vertex (active), right mastoid (reference) and left mastoid (ground). The ABR responses were measured with a tone burst stimulus at 4, 8, 16 and 32 kHz using a computer-based signal averaging system (Tucker-Davis Technology, Alachua, FL, USA), as Yu described previously [37]. The threshold was verified by two unblinded investigators.

**Figure 1:**
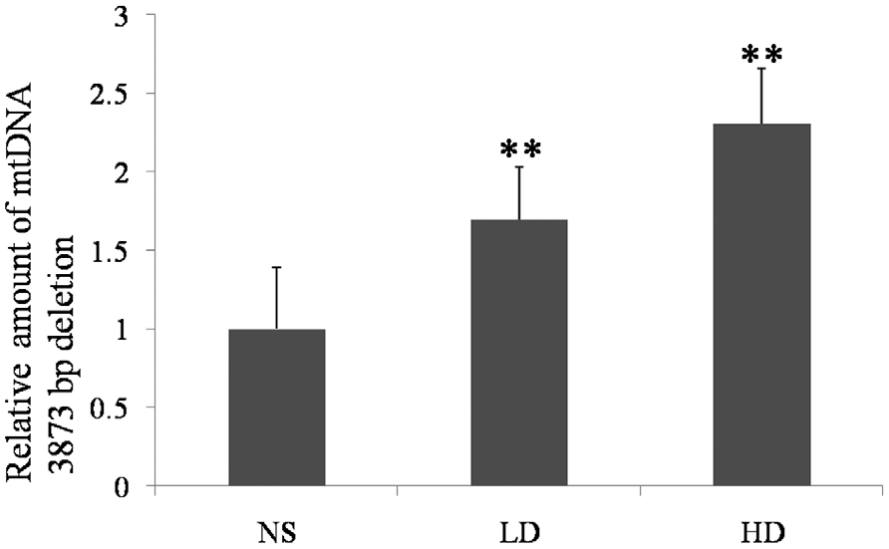
The effect of D-gal on the amount of mtDNA 3873-bp deletion in the cochlear lateral wall of the experimental groups. The accumulation of the mtDNA 3873-bp deletion in the D-gal-treated groups was significantly higher when compared to the control group. (NS, the group treated with normal saline; LD, the group treated with 800 mg/kg D-gal; HD, the group treated with 1000 mg/kg D-gal. **, p<0.01 when compared to the NS group.).

**Figure 2:**
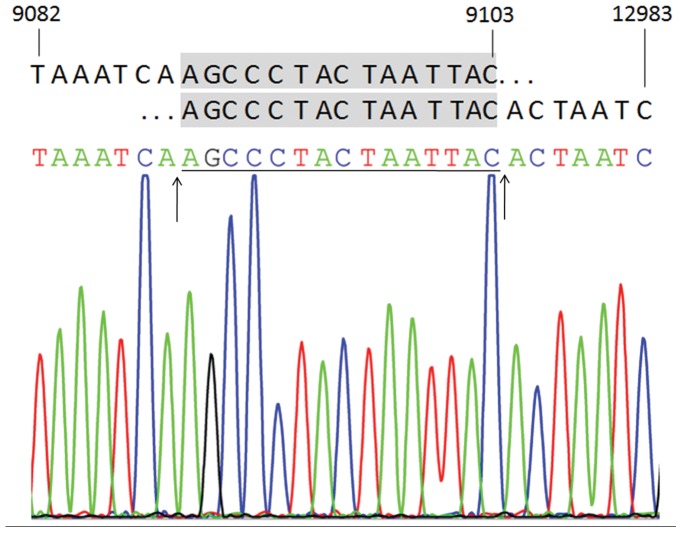
Schematic diagram and sequences of the mtDNA 3873-bp deletion (nt9089–nt12961 or nt9104–nt12976). Bold black letters indicate the nucleotide sequences flanking the breakpoints of the deleted mtDNA, and shaded letters indicate the direct repeats. The sequences of the PCR products are shown below the schematic diagram. The arrowheads point to the possible breakpoints.

### Tissue Sample Preparation

Mice were killed after the last subcutaneous injections. All animals were euthanized under deep anesthesia with chlorpromazine and ketamine hydrochloride, and the temporal bones and plasma were collected. For immunofluorescence experiments, the cochleae from 12 animals (n = 4 from each group) were perfused slowly with cold 4% paraformaldehyde in phosphate-buffered saline (PBS) and then kept in the same fixative at 4°C overnight. Following fixation, the cochleae were decalcified with disodium EDTA. Then, the decalcified cochleae were paraffin embedded, and 5-µm sections were cut and mounted on glass slides for analysis of immunofluorescent labeling. For experiments involving DNA, total RNA and total or mitochondrial protein detection, the cochlear lateral wall, which included the stria vascularis and spiral ligament, was isolated under a stereomicroscope (Zeiss, Germany). Then, the DNA and total RNA from 18 animals (n = 6 from each group) were extracted with a genomic DNA Kit (Tiangen, China) and RNAiso Plus reagent (TaKaRa, Japan), respectively. cDNA was reverse transcribed using a PrimeScript® RT reagent kit (Perfect Real Time; TaKaRa, Japan). The total protein from 18 animals (n = 6 from each group) was isolated with RIPA lysis buffer (Beyotime, China). The mitochondrial proteins from 36 animals (n = 12 from each group) were isolated with the Tissue Mitochondria Isolation Kit (Beyotime, China). Protein concentrations were determined with the BCA Protein Assay Kit (Beyotime, China), with BSA as the standard.

**Figure 3:**
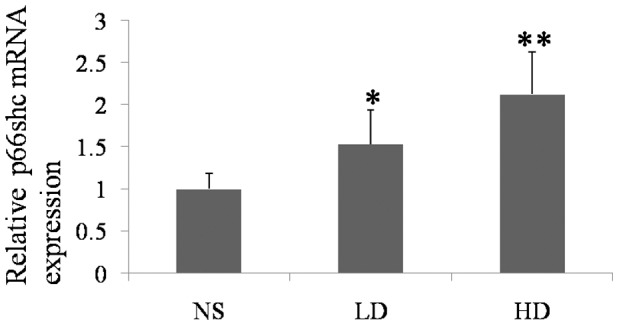
The effect of D-gal on the mRNA levels of p66Shc in the cochlear lateral wall of the experimental groups. With the increasing doses of D-gal, the mRNA levels of p66Shc were increased. (NS, the group treated with normal saline; LD, the group treated with 800 mg/kg D-gal; HD, the group treated with 1000 mg/kg D-gal. *, p<0.05 when compared to the NS group. **, p<0.01 when compared to the NS group.).

### SOD Activity and MDA Assay of Plasma

The SOD (Superoxide Dismutase) activity and MDA (Malondialdehyde) levels in the plasma were quantified with colorimetric kits (Jiancheng, China) according to the manufacturer’s instructions [17].

### Real-time PCR

To evaluate the mtDNA damage induced by D-gal, a sequence designed according to the description of Brossas et al. [38], which represented the new fusion sequence (NFS) that is present in only the deleted mtDNA, was used as a measurement of the mtDNA 3873-bp deletion. Because the D-loop region of mtDNA is rarely deleted, it can represent the conserved segment and be used to determine the copy number of mtDNA. All gene-specific primers used in this study were designed and synthesized at TaKaRa Biotechnology (Dalian) Co. Ltd. and were previously tested for optimal efficiency. The primer pairs for the mtDNA 3873-bp deletion and D-loop were as follows: NFS forward, 5′-CGAAACCACATAAATCAAGCCCTAC-3′; NFS reverse, 5′-AATGATTCGTATGCTGTACATAGCTGTT-3′; D-loop forward, 5′-GGGCTGATTAGACCCGATACCAT-3′; and D-loop reverse, 5′-TACCATCCTCCGTGAAACCAACA-3′. The primer pairs for p66Shc and the internal standard (β-actin) were as follows: p66Shc forward, 5′-CCGACTACCTGTGTTCCTTCTT-3′; p66Shc reverse, 5′-CCCATCTTCAGCAGCCTTTCC-3′; β-actin forward, 5′-CATCCGTAAAGACCTCTATGCCAAC-3′; and β-actin reverse, 5′-ATGGAGCCACCGATCCACA-3′. The DNA and cDNA products were subjected to real-time PCR with the ABI StepOnePlus™ instrument equipped with StepOne software (ABI) for 40 cycles (95°C for 30 s, 95°C for 5 s and 60°C for 5 s) by using the SYBR® Premix Ex Taq™ (Tli RNaseH Plus) kit (TaKaRa, Japan). Each reaction was performed in duplicate and analyzed by melting curves at the end to confirm specificity. The products obtained with primers for detection of the mtDNA 3873-bp deletion were excised from a gel. Then, the products were gel purified, cloned and analyzed using an ABI Prism 377 DNA sequencer (Applied Biosystems, Foster City, CA). The ΔCt method (Ct_deletion_-Ct_D-loop_) was used to calculate the abundance of the mtDNA deletion. The relative amount of the mtDNA 3873-bp deletion and the mRNA levels of p66Shc were calculated with the 2^−**ΔΔCt**^ method.

**Figure 4:**
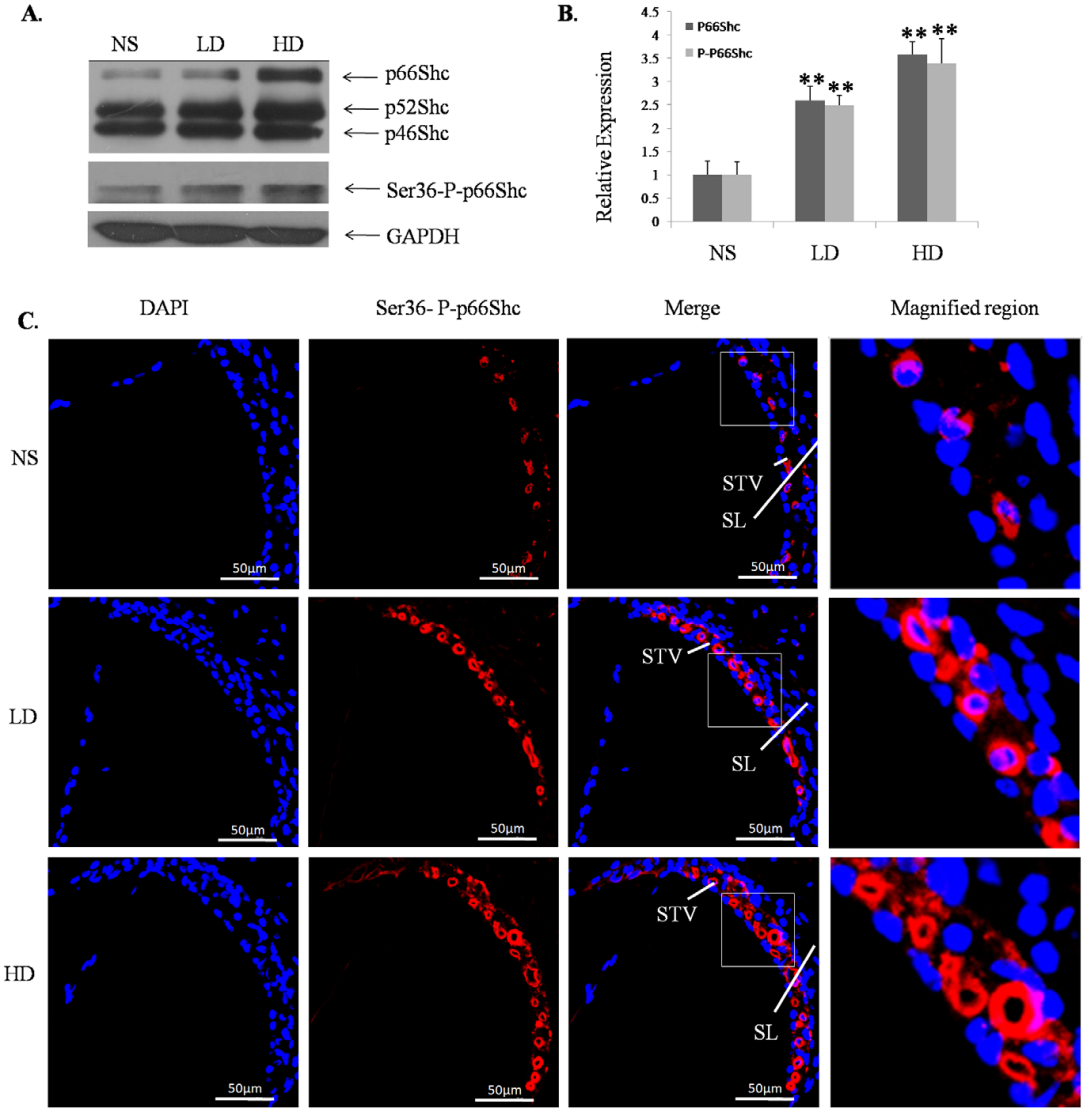
The expression of total SHC protein and Ser36-P-p66Shc in the cochlear lateral wall of the experimental groups. A, Western blot assessment of the protein expression of SHC and Ser36-P-p66Shc in the NS, LD and HD groups. B, Semiquantitative representation of the total protein levels of p66Shc and Ser36-P-p66Shc compared to the control group. C, Cochlea sections were subjected to immunofluorescent labeling of Ser36-P-p66Shc; Ser36-P-p66Shc was mainly expressed in the cytoplasm of cells in the stria vascularis. (NS, the group treated with normal saline; LD, the group treated with 800 mg/kg D-gal; HD, the group treated with 1000 mg/kg D-gal. STV, stria vascularis; SL, spiral ligament. **, p<0.01 when compared to the NS group.).

### Western Blot

We detected the total protein level of p66Shc, p52Shc and p46Shc by using an antibody against SHC (Abcam, USA), which detects the three isoforms simultaneously by binding to a similar domain in all three proteins. The total protein level of SHC and the level of Ser36-P-p66Shc (p66Shc phosphorylated at serine 36) were measured with the corresponding antibodies and normalized to the level of glyceraldehyde-3-phosphate dehydrogenase (GADPH). The mitochondrial p66Shc levels were measured with the SHC antibody and normalized to the voltage-dependent anion channel (VDAC1). For this analysis, 20 µg of protein was separated by 10% or 12% SDS-PAGE and then transferred to nitrocellulose membranes. The SHC and Ser36-P-p66Shc levels were detected after incubation with primary antibodies (SHC, Ser36-P-p66Shc and VDAC1, 1∶1000, Abcam, USA; GAPDH, 1∶5000, Kangchen, China), which was followed by an incubation with a horseradish peroxidase (HRP)-conjugated goat anti-rabbit (1∶5000, Santa Cruz, USA) or goat anti-mouse (1∶2000, Santa Cruz, USA) immunoglobulin, as appropriate. The immunoreactivity was determined using the ECL reaction kit (Beyotime, China) according to the manufacturer’s instructions by exposure on medical film. The band density was quantified with the Image-Pro Plus 6.0 software (Media Cybernetics, Inc., USA).

### Immunofluorescent Labeling

After deparaffinization, rehydration, antigen retrieval and nonspecific antigen site blocking, immunofluorescence was performed on the sections overnight at 4°C with the monoclonal anti-Ser36-P-p66Shc antibody (1∶40, Abcam, USA), which were then incubated for 1 h with the fluorescently tagged secondary antibody. Control staining was performed without the primary antibody. Images were taken with a laser scanning confocal microscope (Nikon, Japan).

### Statistical Analysis

All data are presented as the mean±standard deviation (Mean±S.D.). Statistical analyses were performed with the SPSS 13.0 software (IBM, Armonk, NY, USA). One-way analysis of variance (ANOVA) was used for comparisons of the relative expression levels for the different groups. The least significant difference (LSD) post-hoc test was used to compare differences between two of the groups. Differences with a p-value<0.05 were considered to be statistically significant.

**Figure 5:**
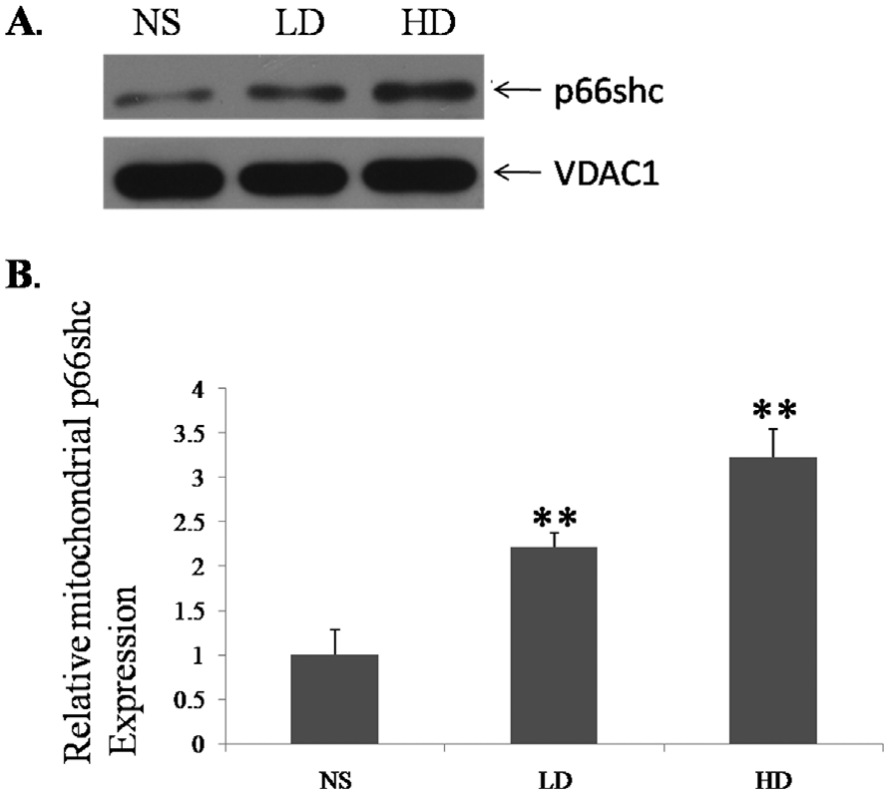
The mitochondrial protein expression of p66Shc in the cochlear lateral wall of the experimental groups. A, Western blot assessment of the protein expression of p66Shc in the NS, LD and HD groups. B, Semiquantitative representation of the relative abundance of the mitochondrial protein levels of p66Shc in D-gal-treated groups compared to the control group. (NS, the group treated with normal saline; LD, the group treated with 800 mg/kg D-gal; HD, the group treated with 1000 mg/kg D-gal. **, p<0.01 when compared to the NS group.).

## Results

### D-gal did not Induce Hearing Loss

To determine the effect of the mtDNA 3873-bp deletion on hearing, the auditory status was evaluated with ABR after the last D-gal administration. There was no significant difference in the ABR threshold (Table 1) among the three experimental groups (p>0.05).

### Decreased SOD Activity and Increased MDA Levels in Plasma Induced by D-gal

The SOD activity in the plasma of mice treated with the low and high doses of D-gal was 170.67±19.11 U/ml and 146.52±13.42 U/ml, respectively, which was significantly lower than that of the control group (198.62±10.35 U/ml) (p<0.01). The MDA levels in the plasma of mice treated with the low and high doses of D-gal were 2.52±0.49 µmol/ml and 3.25±0.47 µmol/ml, respectively, which were significantly higher than that of the control group (1.22±0.20 µmol/ml) (p<0.01) (Table 2).

### Accumulation of the Mitochondrial DNA 3873-bp Deletion Induced by D-gal

The amount of mtDNA 3873-bp deletion was significantly increased in the cochlear lateral wall of the D-gal-treated groups. Compared to the control group, the accumulation of the mtDNA 3873-bp deletion in the low and high dose D-gal groups was increased by approximately 1.70-fold and 2.30-fold, respectively (Fig. 1). The products obtained with the primers for the mtDNA 3873-bp deletion were sequenced to prove the presence of the mtDNA 3873-bp deletion (Fig. 2).

### Increased mRNA Levels of p66Shc Induced by D-gal

The mRNA levels were significantly increased in the cochlear lateral wall of the D-gal-treated groups (Fig. 3). Compared to the control group, the relative expression levels in the low and high dose D-gal-treated groups were increased approximately 1.53-fold and 2.12-fold, respectively.

### Increased Total Protein Levels of p66Shc and Ser36-P-p66Shc Induced by D-gal

The result showed that the total protein level of p66Shc was increased in the cochlear lateral wall of the D-gal-treated groups. However, the relative changes of the total protein levels of p52Shc and p46Shc between the groups were not statistically significant (p>0.05). The levels of Ser36-P-p66Shc were increased in the cochlear lateral wall of the D-gal-treated groups (Fig. 4A and B). Compared to the control group, the total protein levels of p66Shc in the low and high dose groups were increased approximately 2.59-fold and 3.58-fold, respectively, and the levels of Ser-P-p66Shc were increased approximately 2.48- and 3.39-fold, respectively.

In addition, we analyzed the location of the Ser36-P-p66Shc expression by using immunofluorescent labeling. We found that Ser36-P-p66Shc was mainly expressed in the cytoplasm of the cells in the stria vascularis, and it was increased with the increasing doses of D-gal (Fig. 4C).

### Increased Mitochondrial Protein Level of p66Shc Induced by D-gal

To detect the protein levels of p66Shc in the mitochondria, we utilized western blot analysis. The mitochondrial protein levels of p66Shc were significantly increased in the cochlear lateral wall of the D-gal-treated mice (Fig. 5A). Compared to the control group, the mitochondrial protein levels of p66Shc in the D-gal-treated groups were increased approximately 2.21-fold and 3.22-fold in the low and high dose groups, respectively (Fig. 5B).

## Discussion

According to the oxidative stress theory by Denham Harman, the age-related loss of physiological function is due to the progressive accumulation of oxidative damage, which ultimately determines the lifespan of an organism [39]. In this study, we used an overdose of D-gal that was thought to result in the generation of superoxide anions and oxygen-derived free radicals and the formation of advanced glycation end products (AGEs) [40]–[42], which triggers excess ROS and abnormally high oxidative stress, to accelerate the aging of mice. We demonstrated that the plasma SOD activity was decreased and the MDA level in plasma was increased in the D-gal-treated mice. The changes of these two age-related biochemical markers were similar to those that occur during the natural aging process in humans and other animals [43]–[45]. The CD is an important biomarker of aging [46]–[48]. Our previous study on the D-gal-induced rats that mimic aging reported that the amount of CD in the inner ear of the high dose D-gal group was increased 3.69-fold compared to the control group [18]. Zhang et al. [13] reported that the level of the CD in the inner ear of 17- to 19-month-old mice was increased 3.32 fold compared to 7- to 10-month-old mice. In this study, we demonstrated that the amount of CD in the cochlear lateral wall of the high dose D-gal group was increased 2.30-fold compared to the control group. These data indicated that we established a mouse model of aging in the inner ear by using D-gal, which was similar to the D-gal-induced inner ear aging rat model. Corresponding to our previous study in rats [16], [17], [19], there was no significant difference in the ABR threshold among the three experimental groups. The result of the mouse aging model further confirmed the result from the rat aging model that, below a certain level, the mtDNA deletion does not directly lead to a hearing impairment [16], [17], [19].

Most of the p66Shc protein is distributed throughout the cytosol, while a fraction of p66Shc localizes within the inner membrane and intermembrane space of the mitochondria; additionally, oxidative stress stimulates an increase in the mitochondrial p66Shc level [23], [49]–[51]. Protein kinase C β (PKCβ) is activated by oxidative stress and phosphorylates p66Shc on the Ser36 residue, which triggers its translocation to the mitochondria [52], [53]. Our data showed that the mRNA and total protein levels of p66Shc and levels Ser36-P-p66Shc were significantly increased in the cochlear lateral wall of the D-gal-induced aging mice. High amounts of Ser36-P-p66Shc indicate a high activation of the PKCβ pathway and higher oxidative stress in a tissue [54], [55]. Thus, the increased levels of Ser36-P-p66Shc indicated higher oxidative stress in the cochlea lateral wall of the D-gal-induced aging mice. Furthermore, we analyzed the location of the Ser36-P-p66Shc expression in the cochlear lateral wall by using immunofluorescent labeling. The results showed that Ser36-P-p66Shc was mainly expressed in the cytoplasm of the cells in the stria vascularis, which also indicates that there is higher oxidative stress in the stria vascularis. Migliaccio et al. [25] reported that their study of mouse embryo fibroblasts showed that p66Shc was activated upon exposure to agents that induce oxidative stress. Our results further established a positive correlation between the levels of oxidative stress and the amount of p66Shc in the cochlear lateral wall of mice. Consistent with the Ser36-P-p66Shc level, which was increased in the D-gal-induced aging mice, the mitochondrial protein levels of p66Shc were also increased. This result indicates that p66Shc was translocated to the mitochondria in the D-gal-induced aging mice. Pinton et al. [56] reported that phosphorylation of p66Shc at Ser36 triggers a mitochondrial accumulation of p66Shc. It was reported that before Ser36-P-p66Shc is translocated to the mitochondria, it is isomerized by the peptidyl-prolyl isomerase Pin1 and then recognized and dephosphorylated by the phosphatase PP2A [56], [57]. Thus, mitochondrial p66Shc is unphosphorylated.

In the intermembrane space of the mitochondria, p66Shc oxidizes Cytc, resulting in the generation of H_2_O_2_ via the transfer of electrons from the reduced Cytc to oxygen and, thus, increasing the intracellular ROS level [23], which has been suggested to lead to the accumulation of the mtDNA 3873-bp deletion [58]. Trinei et al. [59] reported that the mtDNA 3873-bp deletion was detected in liver samples of wildtype mice, while it was barely detectable in matched tissues from p66Shc^−/−^ mice. This result indicates that the p66Shc expression was correlated with the level of the mtDNA 3873-bp deletion. Therefore, our data indicating that oxidative stress caused an increased expression of p66Shc and Ser36-P-p66Shc and the translocation of p66Shc to the mitochondria may be associated with the accumulation of the mtDNA 3873-bp deletion in the inner ear.

In conclusion, we established a D-gal-induced inner ear aging mouse model in this study and demonstrated the accumulation of the mtDNA 3873-bp deletion in the lateral wall of the D-gal-treated mice, which may be associated with an oxidative stress-related increase in the expression of p66Shc and Ser36-P-p66Shc and the translocation of p66Shc to the mitochondria. Considering that oxidative stress has been associated with numerous human aging processes in addition to presbycusis, the role of p66Shc as a signaling agent may have important therapeutic implications.
